# Perspective of retinal *Chlamydia pneumoniae* infection in Alzheimer’s disease pathogenesis

**DOI:** 10.1186/s13024-026-00964-y

**Published:** 2026-06-24

**Authors:** He Baixu, Wang Qing, Xie Chunming

**Affiliations:** 1 Department of Neurology, Affiliated ZhongDa Hospital, School of Medicine, Jiangsu Key Laboratory of Brain Science and Medicine, Southeast University, Nanjing, Jiangsu 210009 China; 2 Neuropsychiatric Institute, Affiliated ZhongDa Hospital, Southeast University, Nanjing, Jiangsu 210009 China; 3 The Key Laboratory of Developmental Genes and Human Disease, Southeast University, Nanjing, Jiangsu 210009 China

**Keywords:** Alzheimer's disease, *Chlamydia pneumoniae*, Retina, NLRP3 inflammasome, ATN framework

Alzheimer’s disease (AD) is one of the most common neurodegenerative disorders worldwide, from which over 55 million individuals suffer nowadays [1]. Currently biological diagnosis relies on the ATN framework, which is composed of β-amyloid (Aβ), tau, and neurodegeneration [2]. However, large-scale implementation has long been constrained by a variety of limitations, such as invasive procedures, lack of specialized equipment, and patient reluctance [3]. These constraints have fueled people’s interests in exploring alternative, minimally invasive biomarkers, and pathological indicators-including markers of chronic infection and neuroinflammation-which can not only reflect disease progression but also indicate complementary pathogenic mechanisms that operate alongside, or partially independent of protein aggregation [4].

Anti-amyloid monoclonal antibodies in existing therapeutics represent an important advance in disease modification by enabling effective clearance of cerebral Aβ deposition. However, their clinical utility is tempered by safety concerns. A 2023 meta-analysis revealed that these treatments are associated with accelerated brain atrophy, with 21%–37% of participants developing amyloid-related imaging abnormalities (ARIA) [5]. Although the therapeutic rationale of targeting amyloid pathology is not undermined, such observations have catalyzed an exploration of complementary molecular pathways–including neuroimmune mechanisms and infection-inflammation axes–that may inform the development of safer and holistic therapeutic approaches.

Gaire and his colleagues have recently advanced our understanding of retina-brain parallelism in AD pathogenesis [6]. In a comprehensive study of 104 postmortem cases spanning the full AD clinical spectrum, they identified *Chlamydia pneumoniae *(*Cpn*) inclusions in both retinal and cortical tissues, and revealed this pathogen is not merely confined to the AD brain [7]. The authors reported a robust correlation of bacterial burden between retinal and cortical tissues (*r* = 0.62, *p* = 0.014), supporting the idea of the retina as a window for central nervous system (CNS) infection [3]. Quantitative analysis demonstrated a 2.9-fold increase in retinal bacterial load in individuals with AD dementia compared to controls, which was positively associated with apolipoprotein E (APOE) ε4 carrier status and Braak stage, and negatively correlated with Mini-Mental State Examination (MMSE) scores (*r* = − 0.53, *p* < 0.0001) [6]. These observations suggest that retinal infection burden may serve as a quantifiable biomarker of cerebral pathology severity.

This study further explores pathogen-driven mechanisms of inflammasome activation that may define disease-relevant pathways. Whereas NLRP3 activation has traditionally been viewed as primarily being driven by Aβ aggregation [8], this work indicates that *Cpn* infection may also serve as an additional, parallel neuroimmune signal that cooperates with proteinopathy to promote inflammasome assembly and pyroptosis, and the findings also suggest that the infection may potentially function as a disease amplifier [9]. In SH-SY5Y cells, bacterial exposure induced a 3.5-fold increase in Aβ42 accumulation concomitant with NLRP3 activation and lactate dehydrogenase release, suggesting that infection may independently induce neurotoxicity while promoting Aβ deposition. Following intranasal inoculation, transgenic mice exhibited cerebral bacterial colonization with upregulated NLRP3 and interleukin-1 beta (IL-1β) transcription. Six-month chronic infection produced visuo-cognitive deterioration and a 1.6-fold increase in cortical plaque burden, consistent with the hypothesis that chronic infection may amplify core AD pathologies. Notably, the phagocytic index of *Cpn*-associated microglia decreased by 61% in AD retina [6], suggesting that impaired Aβ clearance combined with persistent infection could constitute a critical node of pathological amplification. A schematic summary of these pathological connections is provided in Fig. 1. Nevertheless, these underlying mechanisms remain incompletely understood. Key questions include whether bacterial persistence and Aβ deposition establish bidirectional reinforcement, what signaling cascade involving pathogen-associated molecular patterns primes NLRP3, what role mitochondrial damage and potassium efflux play, and how microglial phagocytic machinery is specifically disrupted.

Therapeutic implications warrant careful consideration. Unlike anti-Aβ monoclonal therapies, which carry risks of ARIA and brain volume loss, pathogen-targeted and inflammasome-modulating strategies represent theoretically promising alternatives. The authors cite epidemiological evidence from a nationwide Taiwanese cohort suggesting that appropriate antibiotic treatment for *Cpn* is significantly associated with reduced risk of AD [10]. However, this observed association does not establish efficacy, and prior antibiotic trials in neurodegeneration have yielded inconsistent results. Therefore, NLRP3 signal suppression or early antibiotic intervention should be regarded as hypothesis-generating strategies necessitating rigorous randomized testing before considering them as established therapeutic approaches. The finding that chronic infection drives sustained inflammasome activation–rather than transient neuroinflammation–supports the rationale for antimicrobial therapy as a cascade-interrupting strategy.

Clinical translation may proceed along multiple dimensions, though all remain speculative at present. The robust correlation between bacterial burden in retina and cortex suggests the potential for retinal monitoring of cerebral infection [3]. Adaptive optics scanning laser ophthalmoscopy may capture microglial morphological alterations, while aqueous humor samples could provide surrogate markers [6, 10] by way of PCR detection of *Cpn* DNA or enzyme-linked immunosorbent assay (ELISA) quantification of IL-1β levels. Future diagnostic development might target activated caspase-1 or gasdermin D (GSDMD) with localized near-infrared probes to enable molecular imaging of inflammasome activation. Ultimately, randomized controlled trials stratified by APOE genotype and baseline inflammatory markers will be essential to evaluate disease-modifying efficacy of early antibiotic intervention or NLRP3 inhibitors (e.g., MCC950) [10] in targeted therapy of the infection-inflammation axis.

However, these findings are subject to some challenges. First, due to the cross-sectional human data [6], temporal causal inference is not possible. Prospective longitudinal studies are essential to establish the sequence of infection and inflammasome activation in the AD spectrum. Second, the specific molecular mechanisms linking microglial dysfunction to impaired *Cpn* clearance remain poorly defined. Key pathways, including pathogen recognition receptors, phagocytic mechanisms, and inflammasome activation cascades, require systematic functional characterization [9]. Third, the infectious hypothesis of AD remains controversial. *The Infectious Hypothesis of Alzheimer’s Disease: A Comprehensive Review* [11], notes that although multiple independent research groups have detected microbial signatures in brain tissue from AD patients, numerous studies have failed to replicate these findings. This inconsistency may be attributed to methodological heterogeneity. Differences in tissue preservation protocols, variations across diverse detection techniques–including PCR and immunohistochemistry, in situ hybridization and metagenomic sequencing–and inconsistencies in control populations all complicate cross-study comparisons. Moreover, in the context of cross-sectional postmortem studies, it remains difficult to conclude whether neurodegeneration itself creates a permissive environment for the colonization of opportunistic pathogens, or *Cpn* infection initiates the disease. These findings suggest that, although *Cpn* represents a potential bacterial candidate in the infectious hypothesis of AD, capable of exacerbating neurodegeneration through inflammatory and amyloid pathways, the definitive causal role in AD remains unproven, particularly given that the current etiological landscape remains to be fully elucidated.

In summary, Gaire et al. [6] provide compelling evidence that *Cpn* may act as a potential disease amplifier in AD, driving NLRP3-mediated pyroptosis via infection-inflammation mechanisms that may operate, at least in part, independently of protein aggregation [9]. By establishing quantitative retina-brain associations in bacterial burden [3], this work offers theoretical feasibility of noninvasive in vivo monitoring of cerebral infection. By identifying pathogen clearance dysfunction rather than mere infection load as a critical node, it reveals actionable therapeutic targets. However, these findings are preliminary. Future research should focus on longitudinal clinical studies to establish temporal relationships, developing retinal molecular imaging probes for *Cpn* or inflammasome activation biomarkers, and conducting rigorously designed clinical trials of antibiotics or NLRP3 inhibitors. Such efforts may eventually expand therapeutic strategies beyond late-stage protein clearance to encompass early interruption of pathogen-driven neuroimmune injury [4], though it remains to be definitively validated.

**Fig. 1:**
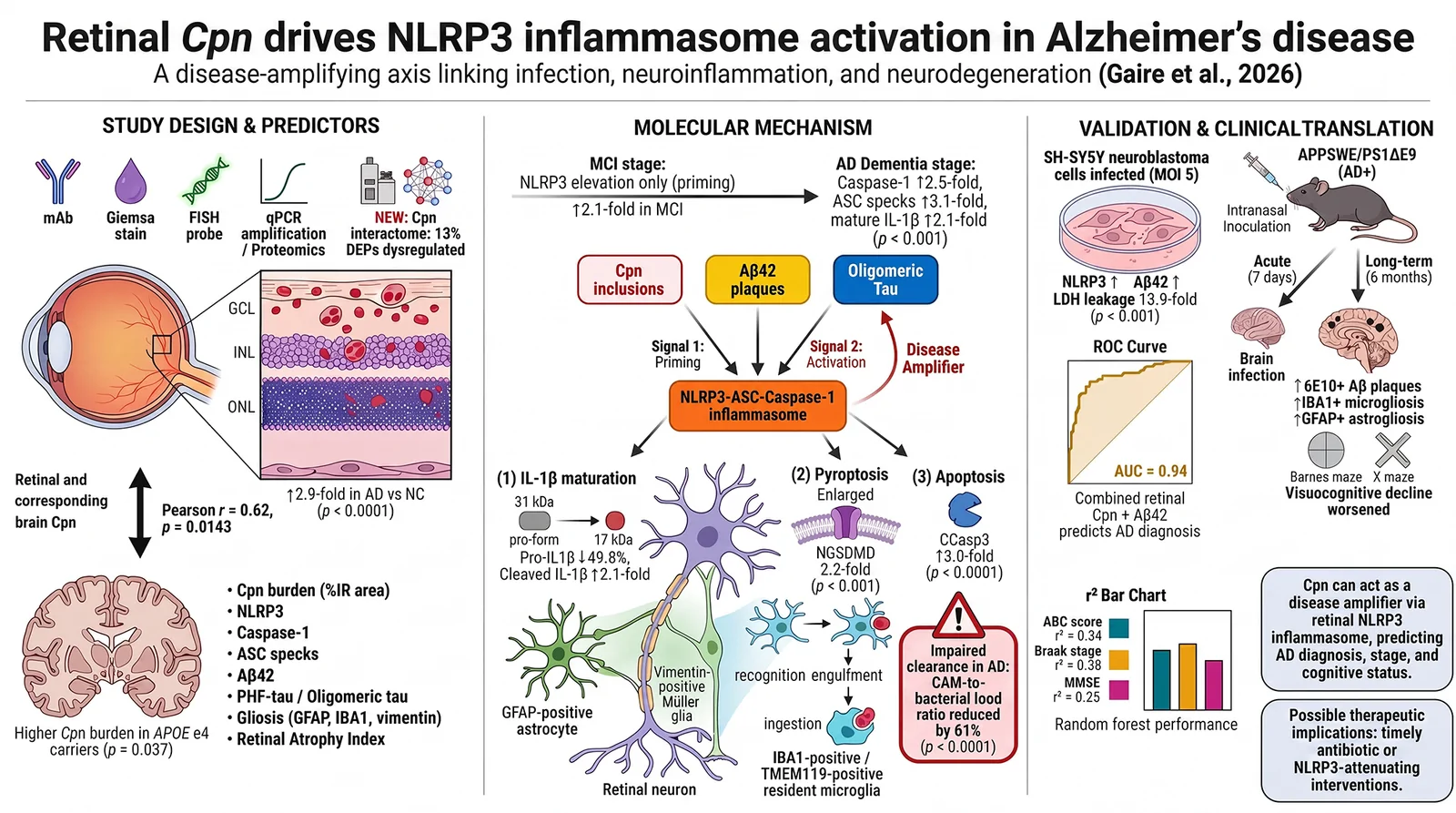
Proposed role of retinal *Cpn* infection in Alzheimer’s disease pathogenesis. The schematic summarizes the proposed role of retinal *Chlamydia pneumoniae* (*Cpn*) infection in promoting neuroinflammation and neurodegeneration in Alzheimer’s disease (AD). Left panel: retinal and corresponding cortical tissues exhibit correlated bacterial burden, with increased *Cpn* load associated with APOE ε4 carrier status, retinal atrophy, gliosis, and AD-related pathological markers. Center panel: *Cpn* infection may function as a priming signal for NLRP3 inflammasome activation, promoting ASC speck formation, caspase-1 activation, IL-1β maturation, pyroptosis, apoptosis, and impaired microglial phagocytic clearance. These processes may synergize with Aβ accumulation and tau oligomerization to amplify AD pathology. Right panel: experimental validation in SH-SY5Y cells and APP/PS1 mice demonstrated increased Aβ42 accumulation, inflammasome activation, cerebral infection, gliosis, and visuo-cognitive decline following chronic infection. The figure further highlights potential translational applications, including retinal molecular imaging, biomarker-based diagnosis, and therapeutic strategies targeting infection-associated inflammasome activation

## Data Availability

No datasets were generated or analysed during the current study.

## Abbreviations

AD: Alzheimer's disease
APOE: Apolipoprotein E
ARIA: Amyloid-related imaging abnormalities
Aβ: Amyloid-beta
Aβ42: Amyloid-beta 42
CNS: Central nervous system
ELISA: Enzyme-linked immunosorbent assay
GSDMD: Gasdermin D
IL-1β: Interleukin-1 beta
LDH: Lactate dehydrogenase
MMSE: Mini-Mental State Examination
NLRP3: NOD-like receptor family pyrin domain containing 3
PCR: Polymerase chain reaction
Cpn: Chlamydia pneumoniae
